# Supplementary material for: Structural basis for the adaptation and function of chlorophyll f in photosystem I
Source: Nat Commun. 2020 Jan 13;11:238. doi: 10.1038/s41467-019-13898-5 (PMC6957486; doi:10.1038/s41467-019-13898-5)
Supplement: Supplementary file 1 — Supplementary Information [file 41467_2019_13898_MOESM1_ESM.pdf]

Supplementary Information

Structural basis for the adaptation and function of chlorophyll *f* in photosystem I

Koji Kato, Toshiyuki Shinoda, Ryo Nagao et al.

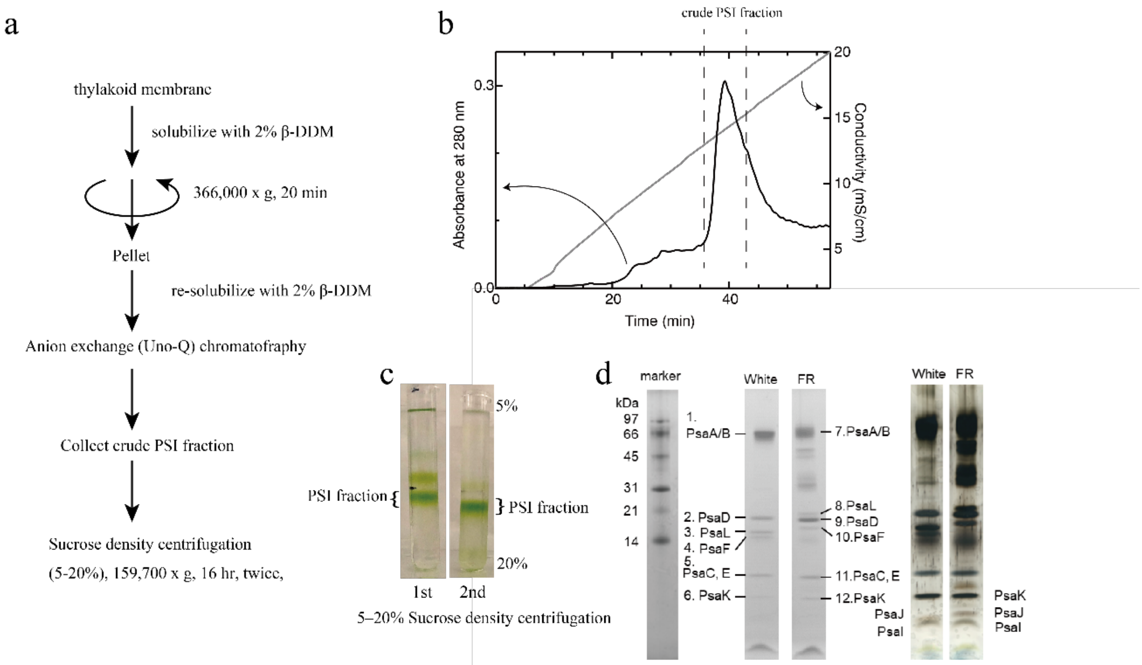

Supplementary Fig. 1. Preparation of PSI trimers. a, Purification procedure for the PSI complex from *H. hongdechloris*. **b,** Elution profile of crude PSI from an anion-exchange column (UnoQ1, BioRad). The UnoQ1 column was previously equilibrated with 10% (w/ v) glycerol/ 50 mM MES (pH 6.0)/ 5 mM $CaCl_2$/ 0 M NaCl/ 0.04% DDM. Crude PSI was eluted with a gradient of 0-400 mM NaCl in the same buffer. **c,** Sucrose density gradient centrifugation of the crude PSI fraction. **d,** SDS-PAGE profiles of the purified white PSI and far-red PSI. From left side, molecular marker, stained with CBB (white PSI, far-red PSI), silver stain (white PSI, far-red PSI)

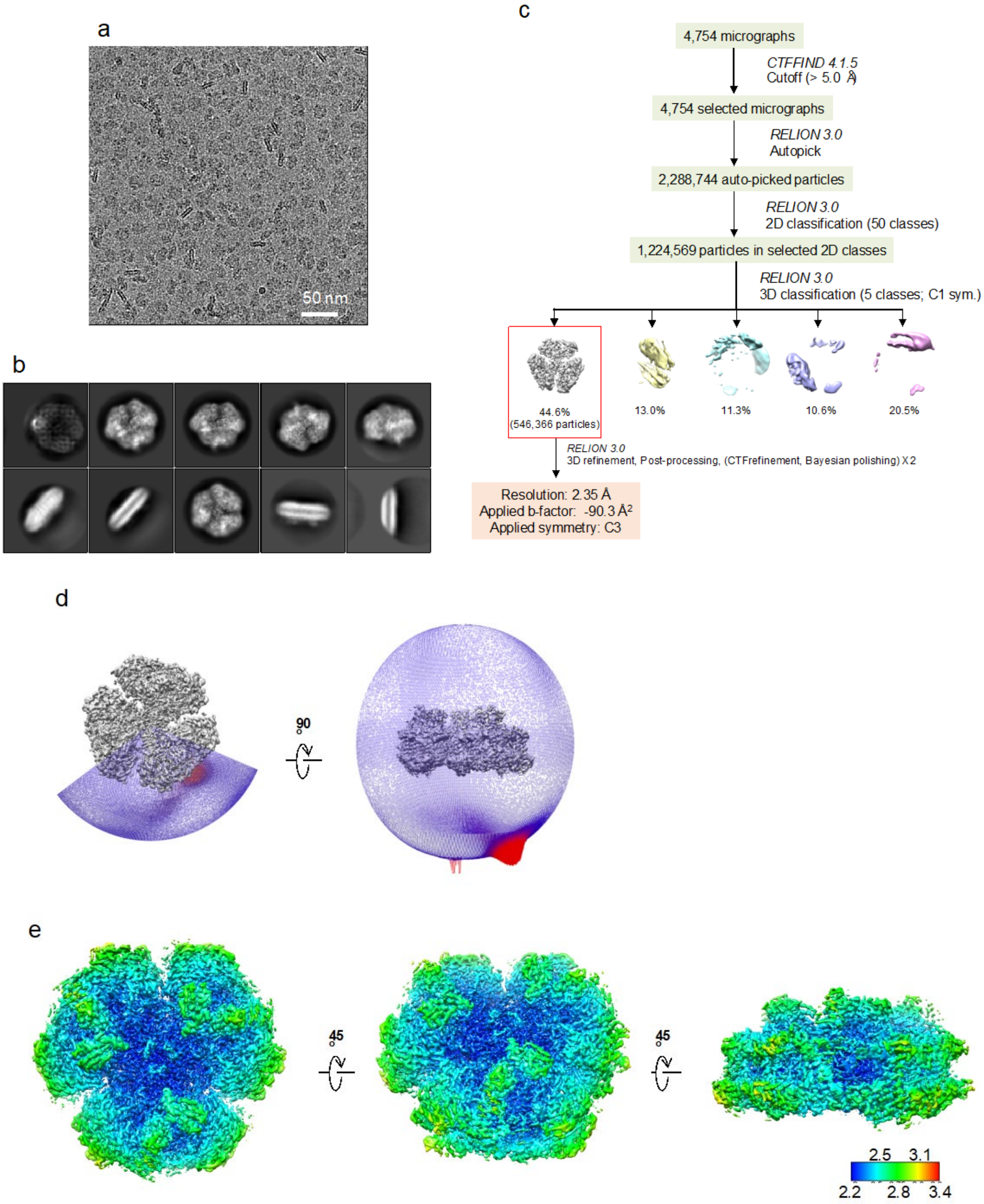
a
50 nm
b
c
4,754 micrographs
CTFFIND 4.1.5
Cutoff (> 5.0 Å)
4,754 selected micrographs
RELION 3.0
Autopick
2,288,744 auto-picked particles
RELION 3.0
2D classification (50 classes)
1,224,569 particles in selected 2D classes
RELION 3.0
3D classification (5 classes; C1 sym.)
44.6%
(546,366 particles)
13.0%
11.3%
10.6%
20.5%
RELION 3.0
3D refinement, Post-processing, (CTFrefinement, Bayesian polishing) X2
Resolution: 2.35 Å
Applied b-factor: -90.3 Å²
Applied symmetry: C3
d
90°
e
45°
45°
2.5 3.1
2.2 2.8 3.4

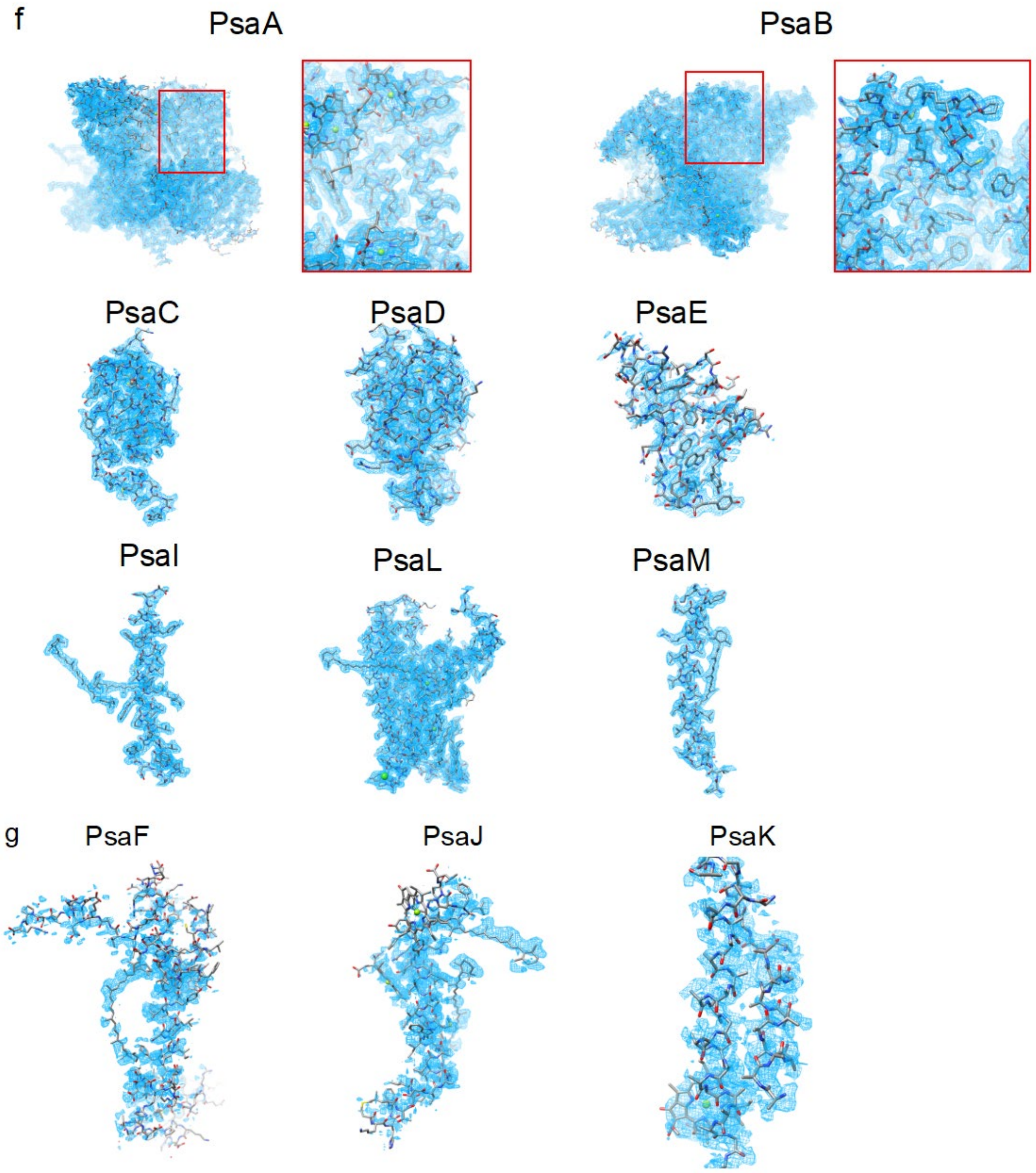

Supplementary Fig. 2. Cryo-EM data collection and processing of the white PSI. a, A representative cryo-EM micrograph of the white PSI trimer. **b,** Representative 2D classes of the PSI trimer particles. **c,** Flowchart for the classification of the white PSI trimer. The white PSI trimer structure was reconstructed at 2.35 Å resolution from 546,366 particles. See Methods section for more details. **d,** Angular distribution of the particles used for reconstruction of the white PSI trimer. Each cylinder represents one view and the height of the cylinder is proportional to the number of particles for that view. **e,** Local resolution maps of the white PSI trimer. **f,** Cryo-EM density maps and structures of the white PSI core subunits that are assigned in the present study. The densities for each subunit of the white PSI are shown as blue meshes and the corresponding models are shown as gray sticks. **g,** Cryo-EM density maps for PsaF, PsaJ and PsaK from the white PSI core superimposed with their structures from *T. elongatus*. The densities for each subunit of the white PSI are shown as blue meshes and the corresponding models are shown as gray sticks.

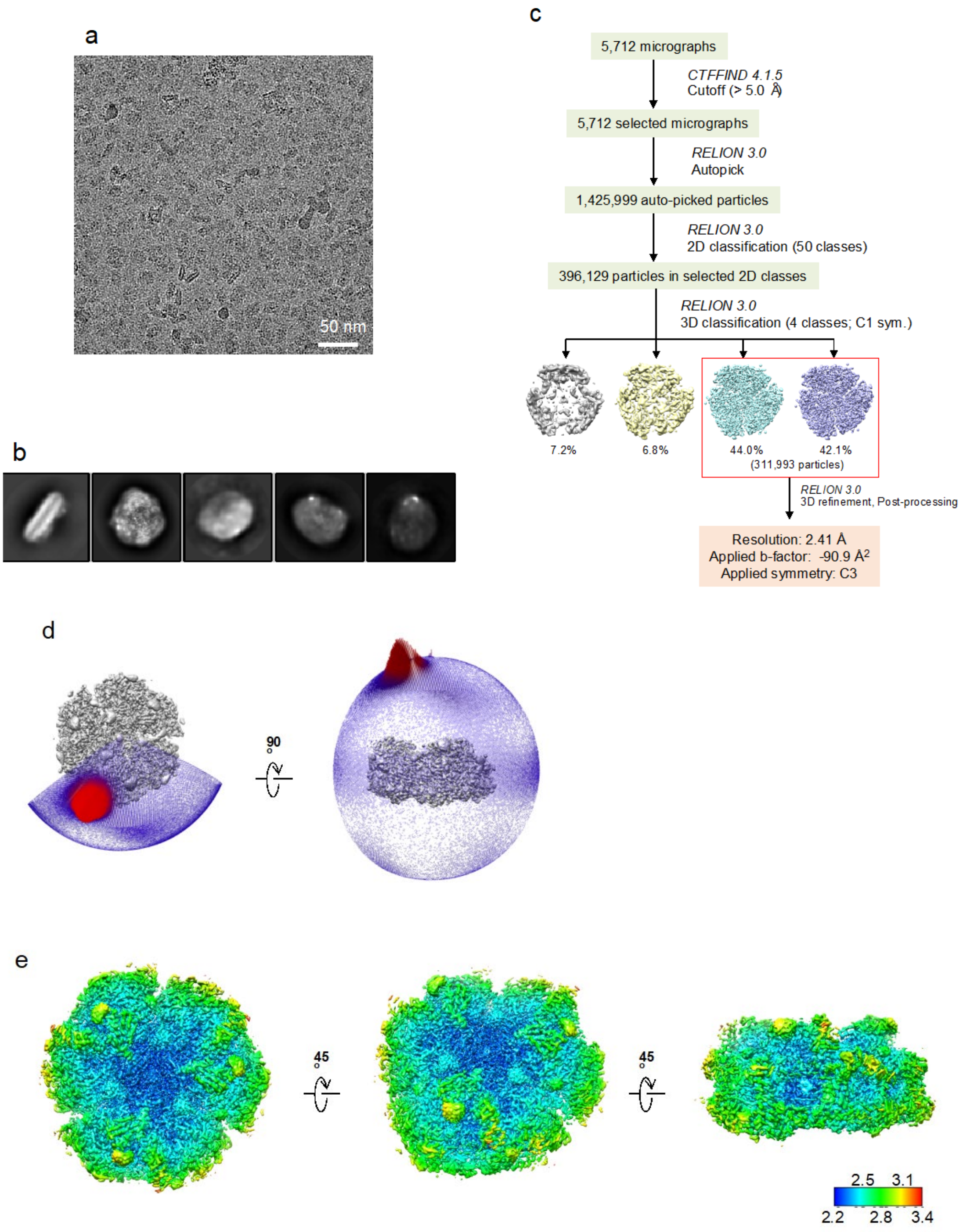
a
50 nm
b
c
5,712 micrographs
CTFFIND 4.1.5
Cutoff (> 5.0 Å)
5,712 selected micrographs
RELION 3.0
Autopick
1,425,999 auto-picked particles
RELION 3.0
2D classification (50 classes)
396,129 particles in selected 2D classes
RELION 3.0
3D classification (4 classes; C1 sym.)
7.2%
6.8%
44.0%
42.1%
(311,993 particles)
RELION 3.0
3D refinement, Post-processing
Resolution: 2.41 Å
Applied b-factor: -90.9 Å²
Applied symmetry: C3
d
90°
e
45°
45°
2.5 3.1
2.2 2.8 3.4

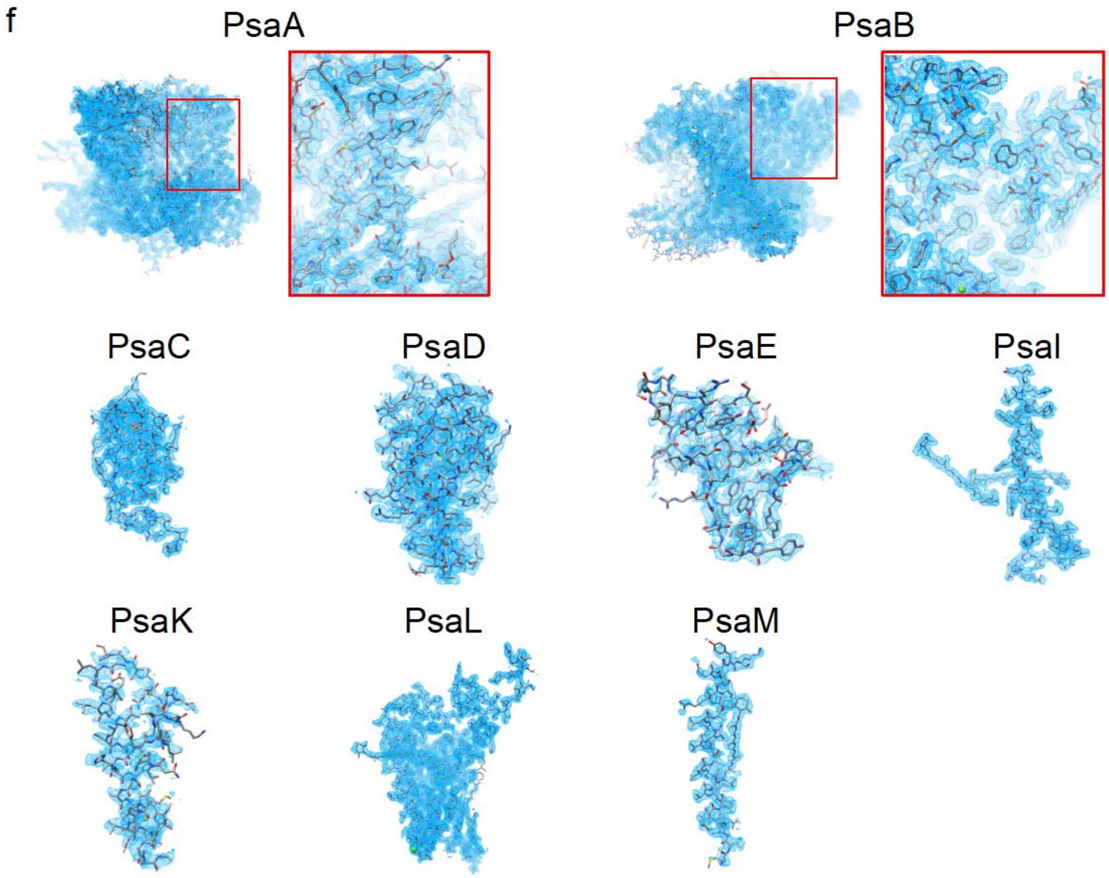

Supplementary Fig. 3. Cryo-EM data collection and processing of the far-red PSI. a, A representative cryo-EM micrograph of the far-red PSI trimer. **b,** Representative 2D classes of the PSI trimer particles. **c,** Flowchart for the classification of the far-red PSI trimer. The far-red PSI trimer structure was reconstructed at 2.41 Å resolution from 311,993 particles. See Methods section for more details. **d,** Angular distribution of the particles used for reconstruction of the far-red PSI trimer. Each cylinder represents one view and the height of the cylinder is proportional to the number of particles for that view. **e,** Local resolution maps of the far-red PSI trimer. **f,** Cryo-EM density maps and structures of the far-red PSI core subunits assigned in the present study. The densities for each subunit of the far-red PSI are shown as blue meshes and the corresponding models are shown as gray sticks.

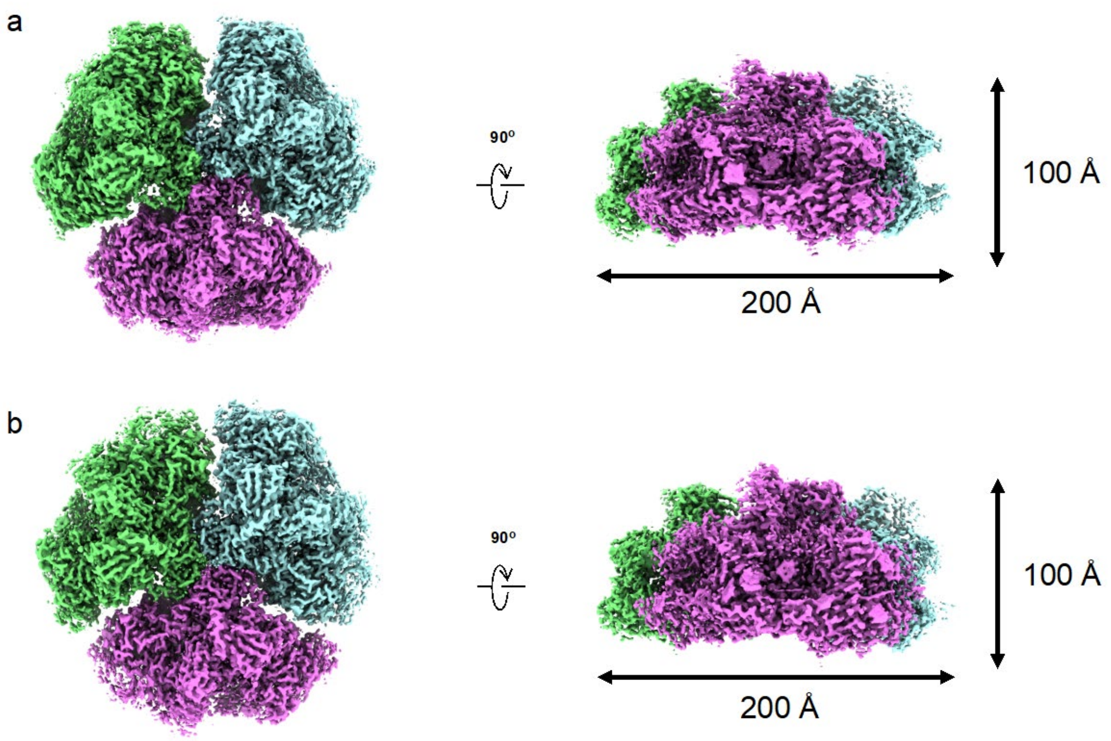

Supplementary Fig. 4. Cryo-EM densities and overall structures of the white PSI and far-red PSI. a, Cryo-EM density map of the white PSI trimer viewed along the membrane normal from the stromal side (Left) and its side view (Right). **b,** Cryo-EM density map of the far-red PSI trimer viewed along the membrane normal from the stromal side (Left) and its side view (Right).

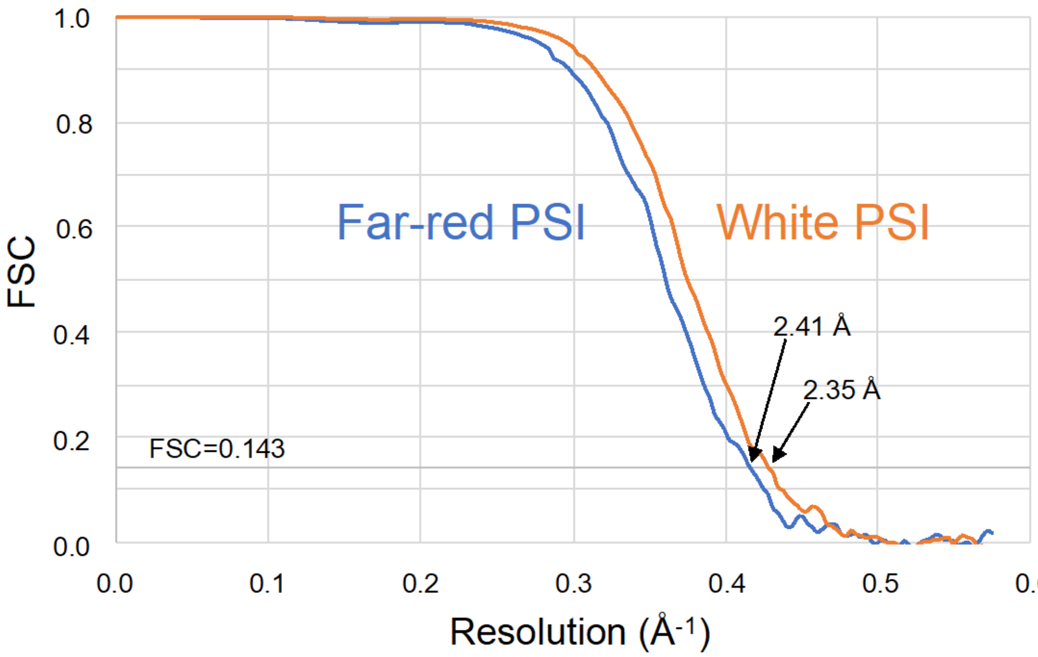

Supplementary Fig. 5. Gold standard fourier shell correlation curves for the white and far-red PSI. FSC curves of the white PSI (orange) and far-red PSI trimers (blue) were calculated between independently refined half maps used for the structure reconstructions.

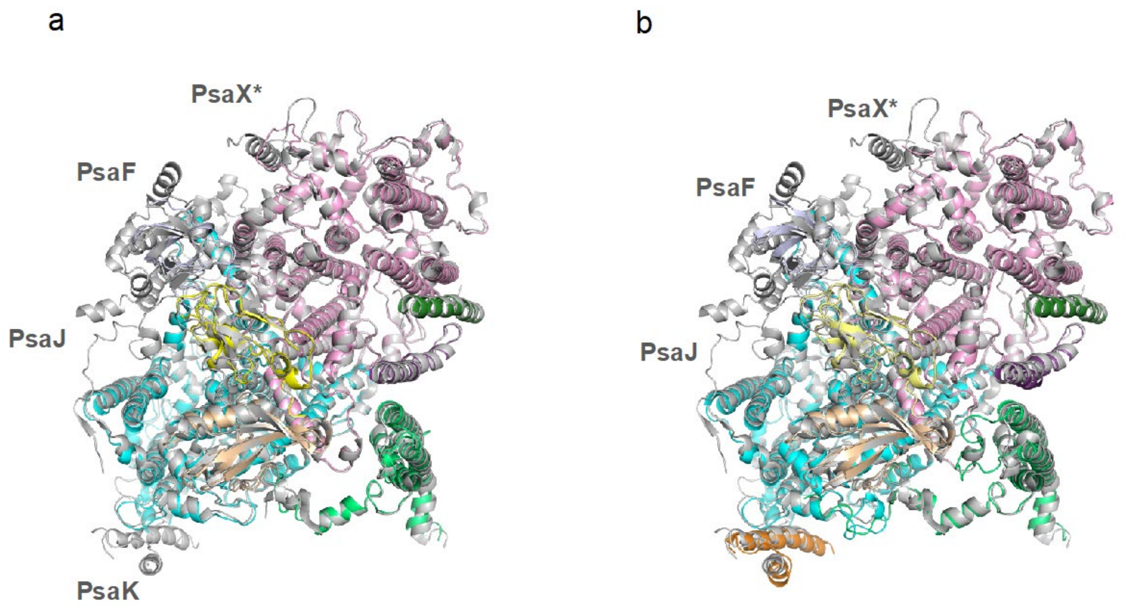

Supplementary Fig. 6. Structure of a white and far-red PSI monomers. a, Superposition of the white PSI monomer (colored) of *H. hongdechloris* with a *T. elongatus* PSI monomer (gray) viewed along the membrane normal from the stromal side. **b,** Superposition of the far-red PSI monomer (colored) of *H. hongdechloris* with a *T. elongatus* PSI monomer (gray) viewed along the membrane normal from the stromal side. Subunits that are not modeled in the structure of *H. hongdechloris* are labeled.

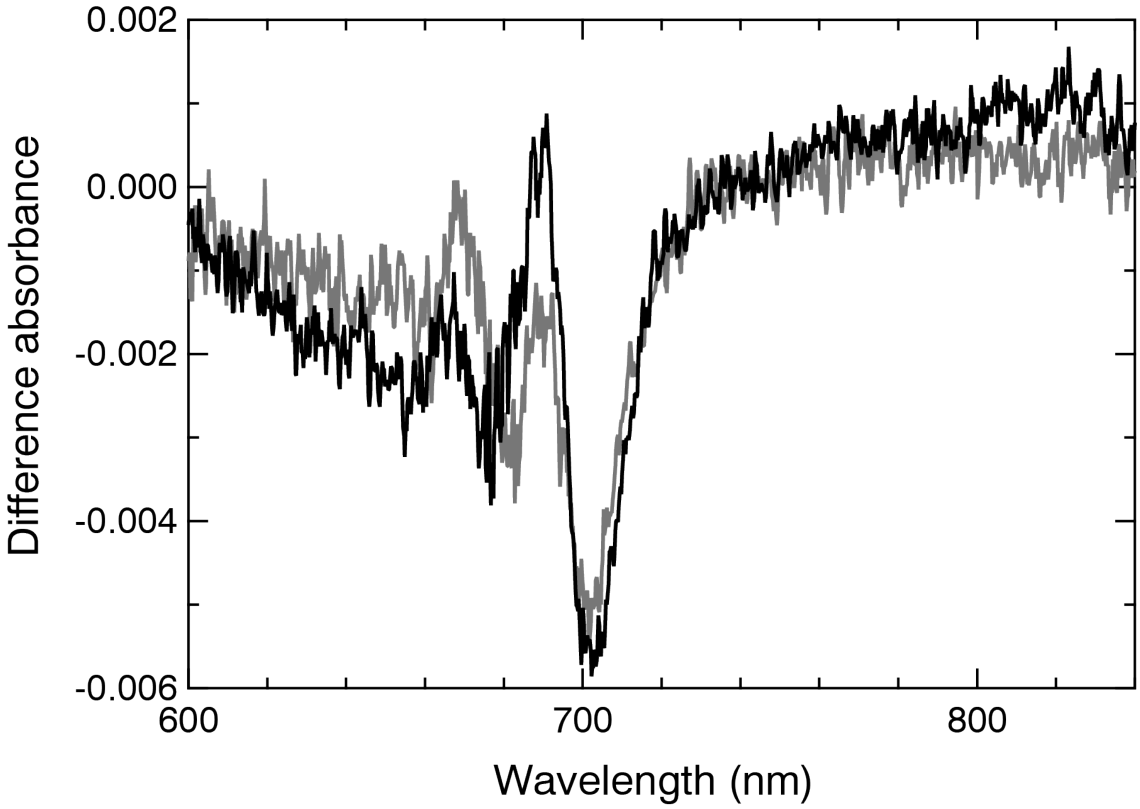

Supplementary Fig. 7. The difference absorption spectra of P700. Light-induced difference absorption spectra of *Themosynechococcus elongatus* PS1 (black line) and far-red PSI of *H. hongdechloris* (gray line). Samples were suspended in 0.025% β-DDM, 1 mM ascorbate, 200 μM methyl viologen, 25 μM *N*,*N*,*N*′,*N*′-tetramethyl-p-phenylenediamine, and 50 mM Tris-HCl (pH 8.0) and were illuminated with blue light. The Qy maximum of each sample was adjusted to 0.9. Both spectra were recorded at room temperature.

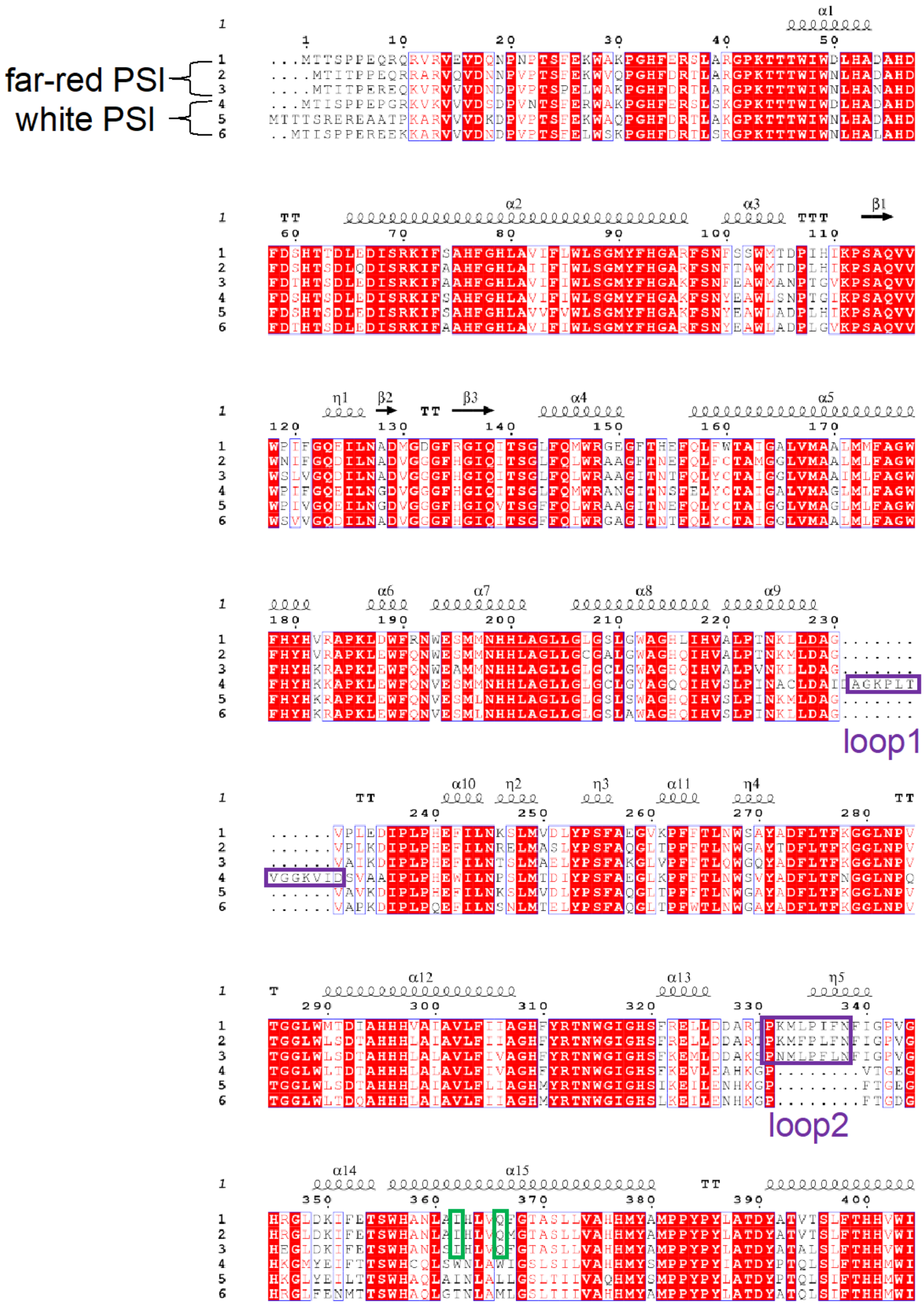
far-red PSI
white PSI
loop1
loop2

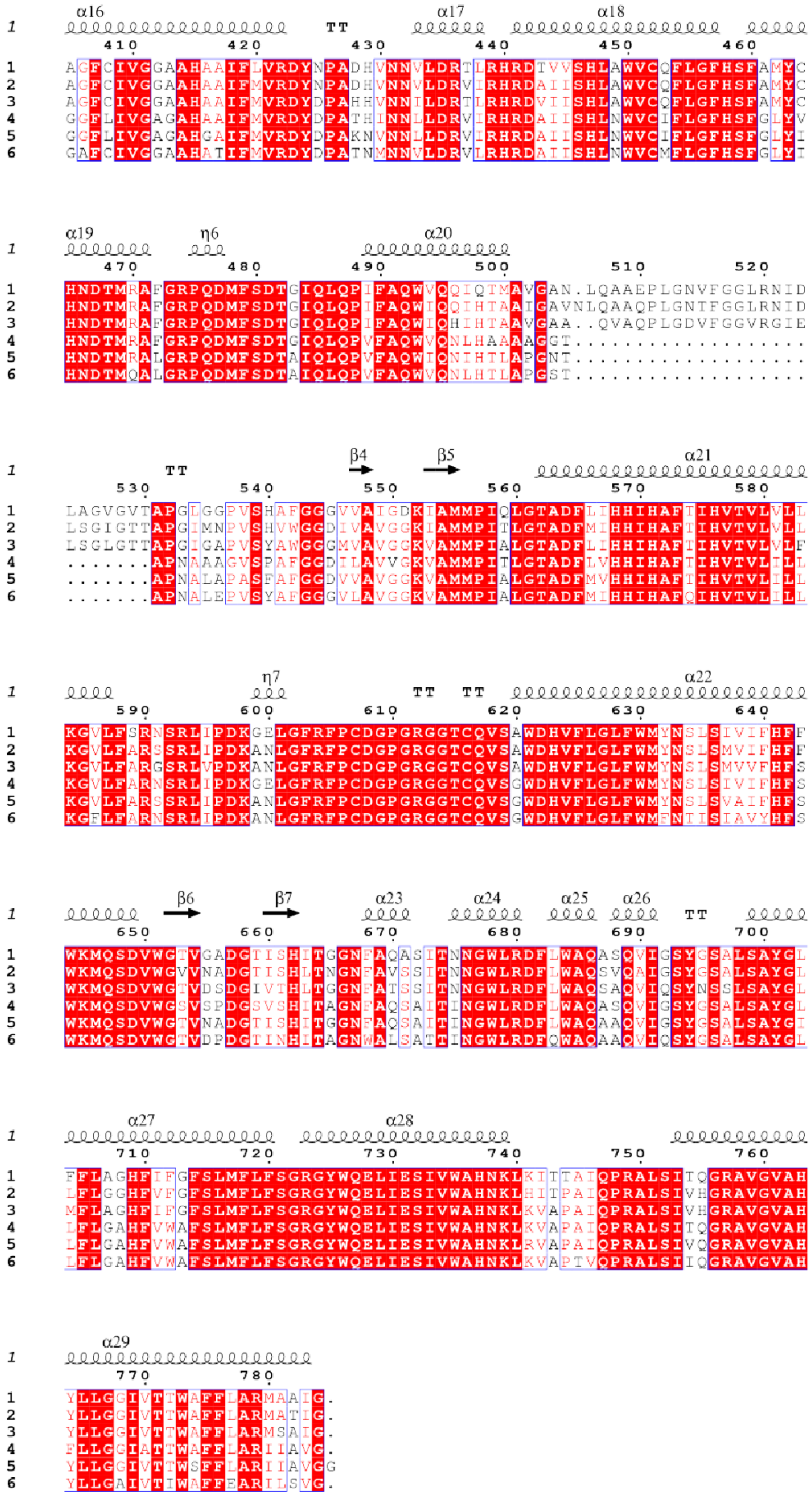

Supplementary Fig. 8. Multiple sequence alignment (ClustalW and ESPript) of the PsaA subunit from three species of cyanobacteria known to undergo remodeling. Secondary structural elements are shown above the sequence. Completely conserved residues are highlighted in red. Loop1 (Ala232-Asp244) and loop2 (Pro331-Asn338) are labeled and indicated with purple boxes. Green boxes show amino acid resiudes related to Chl *f*-binding in the far-red PSI. The cyanobacterial species shown are 1: *H. hongdechloris* PsaA1 (far-red), 2: *Leptolyngbya* sp. strain JSC-1 PsbA2 (far-red), 3: *Chroococcidiopsis thermalis* PCC7203 PsaA2 (far-red), *H. hongdechloris* PsaA2 (white), 5: *Leptolyngbya* sp. strain JSC-1 PsbA1 (white), 6: *Chroococcidiopsis thermalis* PCC7203 PsaA1 (white).

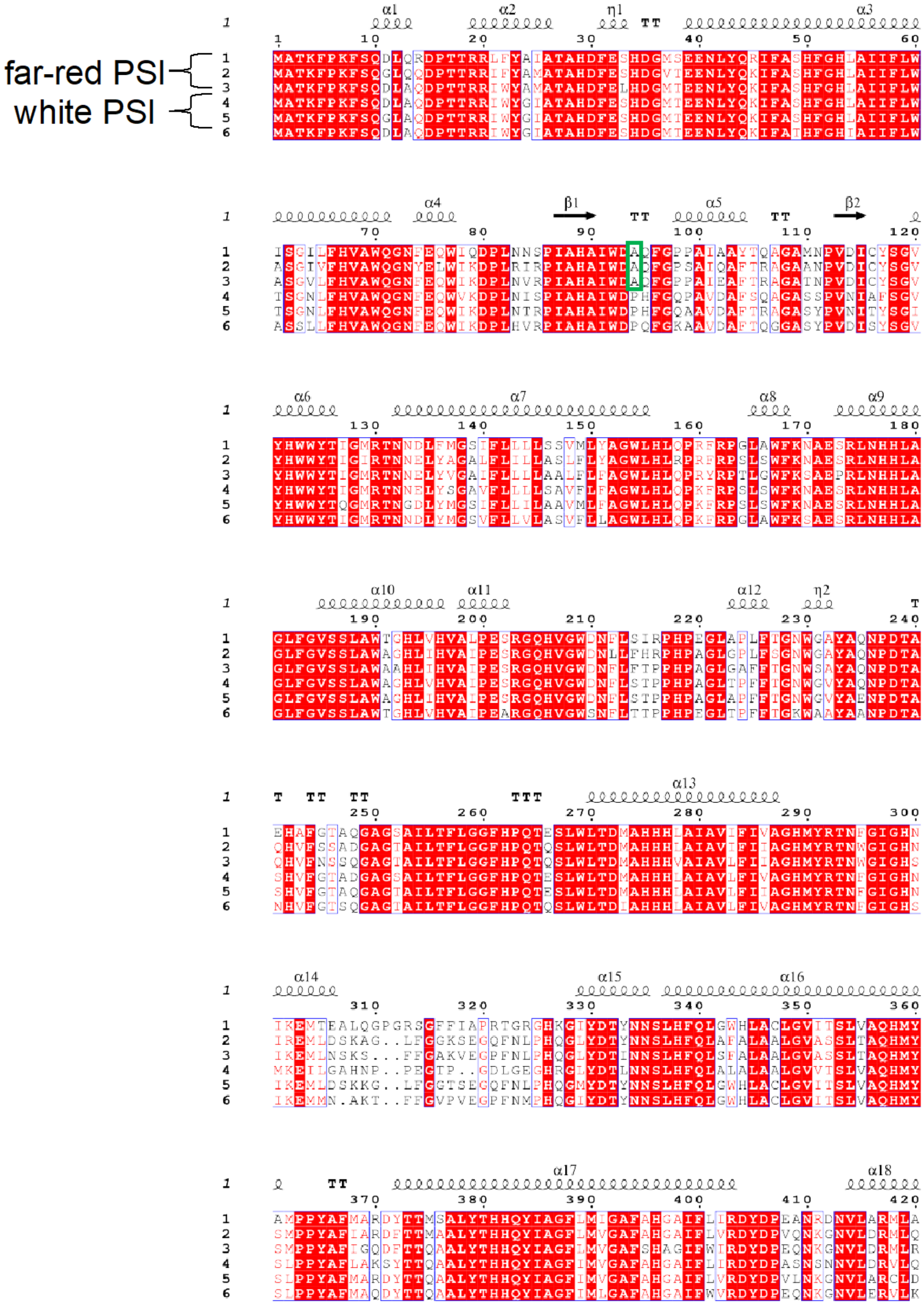
far-red PSI
white PSI

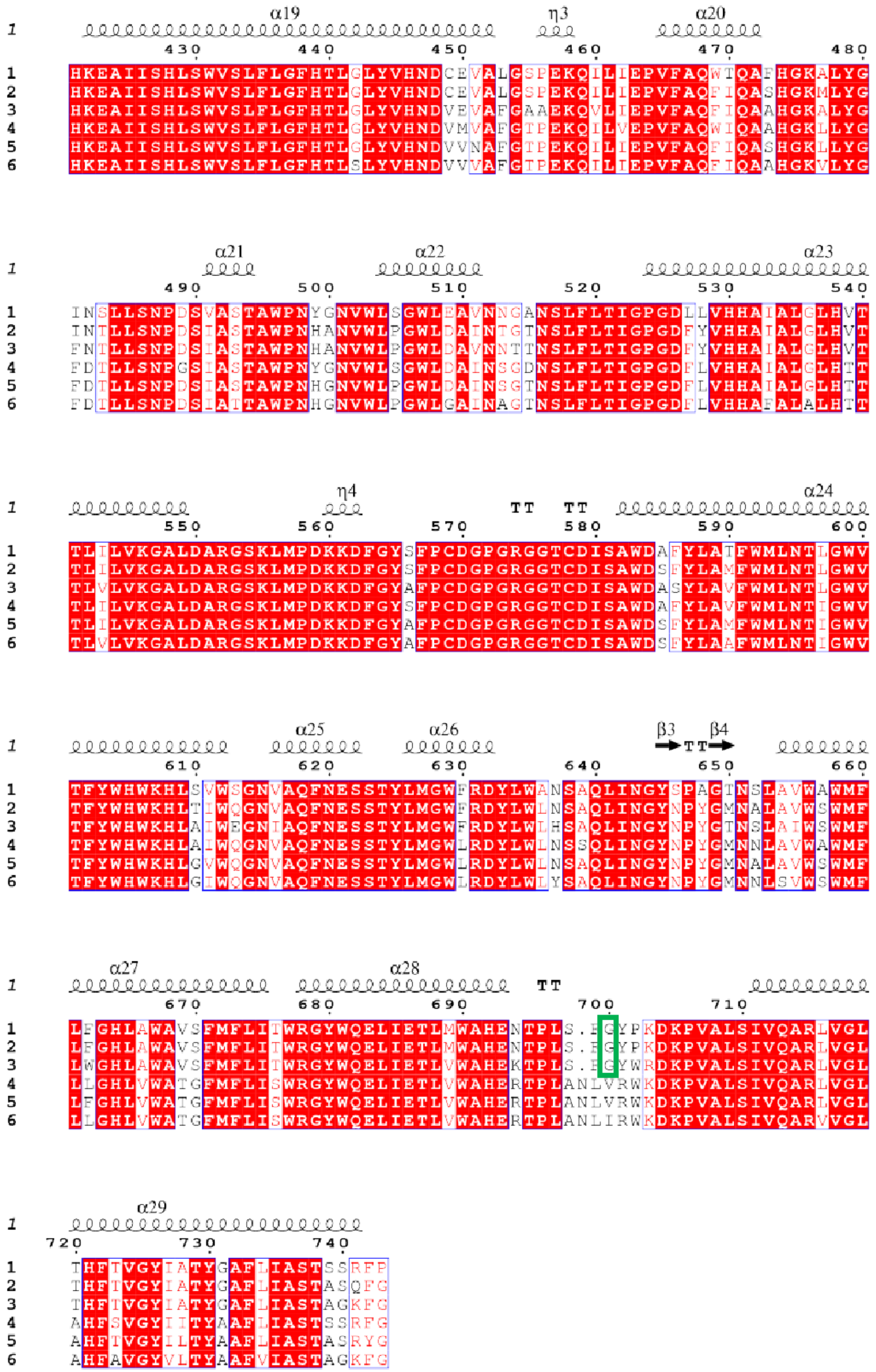

Supplementary Fig. 9. Multiple sequence alignment (ClustalW and ESPript) of the PsaB subunit from three species of cyanobacteria known to undergo remodeling. Secondary structural elements are shown above the sequence. Completely conserved residues are highlighted in red. Green boxes show amino acid residues related to Chl *f*-

binding in the far-red PSI. The cyanobacterial species shown are 1: *H. hongdechloris* PsaB1 (far-red), 2: *Leptolyngbya* sp. strain JSC-1 PsbB2 (far-red), 3: *Chroococcidiopsis thermalis* PCC7203 PsaB2 (far-red), *H. hongdechloris* PsaB2 (white), 5: *Leptolyngbya* sp. strain JSC-1 PsbB1 (white), 6: *Chroococcidiopsis thermalis* PCC7203 PsaB1 (white).

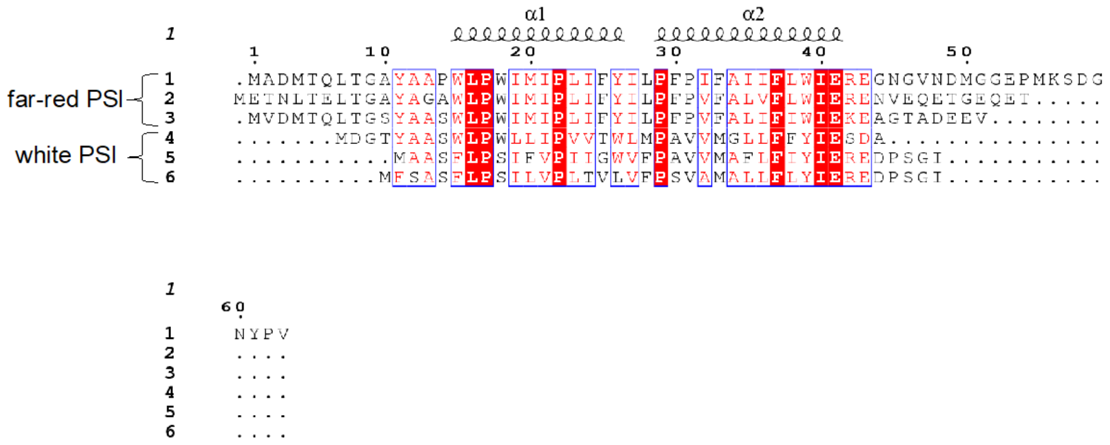

Supplementary Fig. 10. Multiple sequence alignment (ClustalW and ESPript) of the PsaI subunit from three species of cyanobacteria known to undergo remodeling. Secondary structural elements are shown above the sequence. Completely conserved residues are highlighted in red. The cyanobacterial species shown are 1: *H. hongdechloris* PsaI2 (far-red), 2: *Leptolyngbya* sp. strain JSC-1 PsbI2 (far-red), 3: *Chroococcidiopsis thermalis* PCC7203 PsaI (far-red), *H. hongdechloris* PsaI3 (white), 5: *Leptolyngbya* sp. strain JSC-1 PsbI1 (white), 6: *Chroococcidiopsis thermalis* PCC7203 PsaI1 (white).

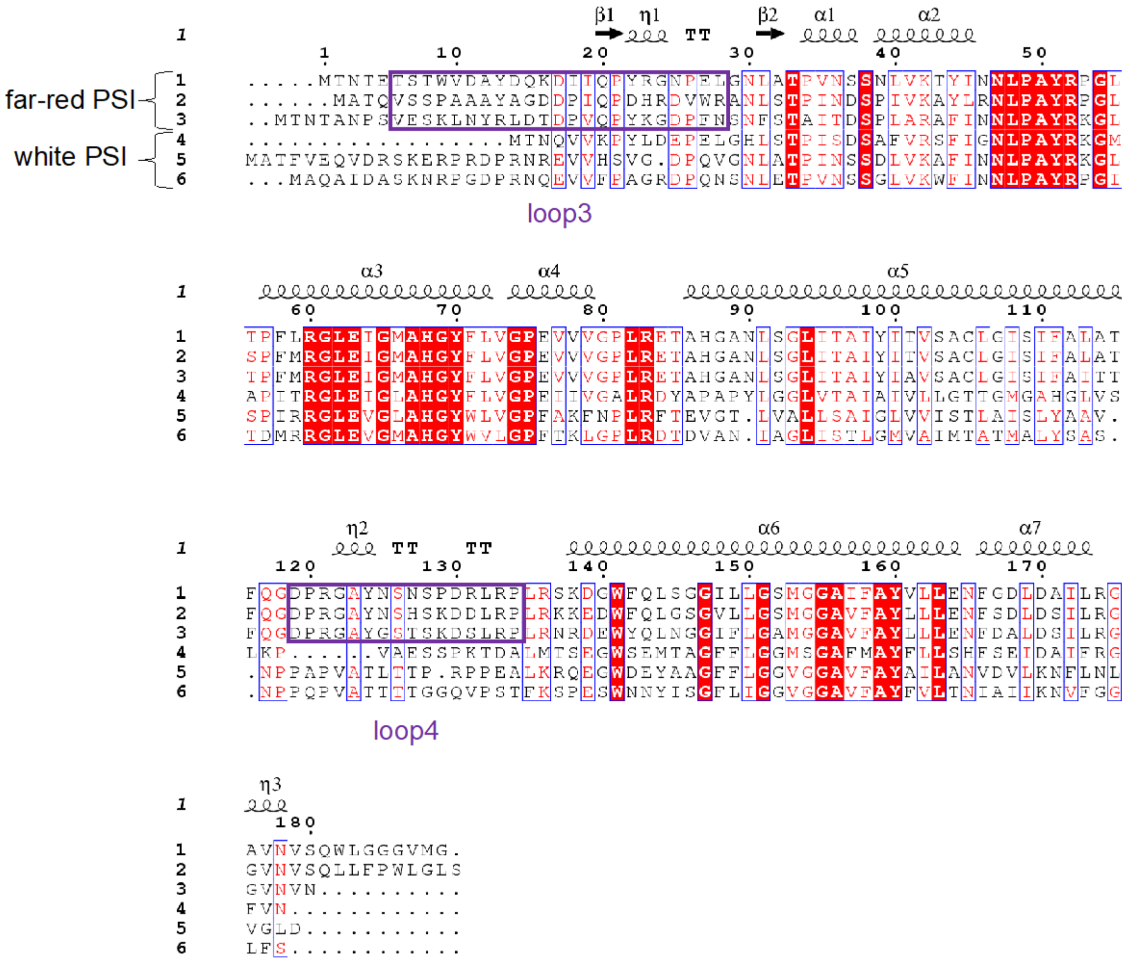

Supplementary Fig. 11. Multiple sequence alignment (ClustalW and ESPript) of the PsaL subunit from three species of cyanobacteria known to undergo remodeling. Secondary structural elements are shown above the sequence. Completely conserved residues are highlighted in red. Loop3 (Thr8-Leu28) and loop4 (Gln117-Pro134) are labeled and indicated with purple boxes. The cyanobacterial species shown are 1: *H. hongdechloris* PsaL1 (far-red), 2: *Leptolyngbya* sp. strain JSC-1 PsbL2 (far-red), 3: *Chroococcidiopsis thermalis* PCC7203 PsaL2 (far-red), *H. hongdechloris* PsaL2 (white), 5: *Leptolyngbya* sp. strain JSC-1 PsbL1 (white), 6: *Chroococcidiopsis thermalis* PCC7203 PsaL1 (white).

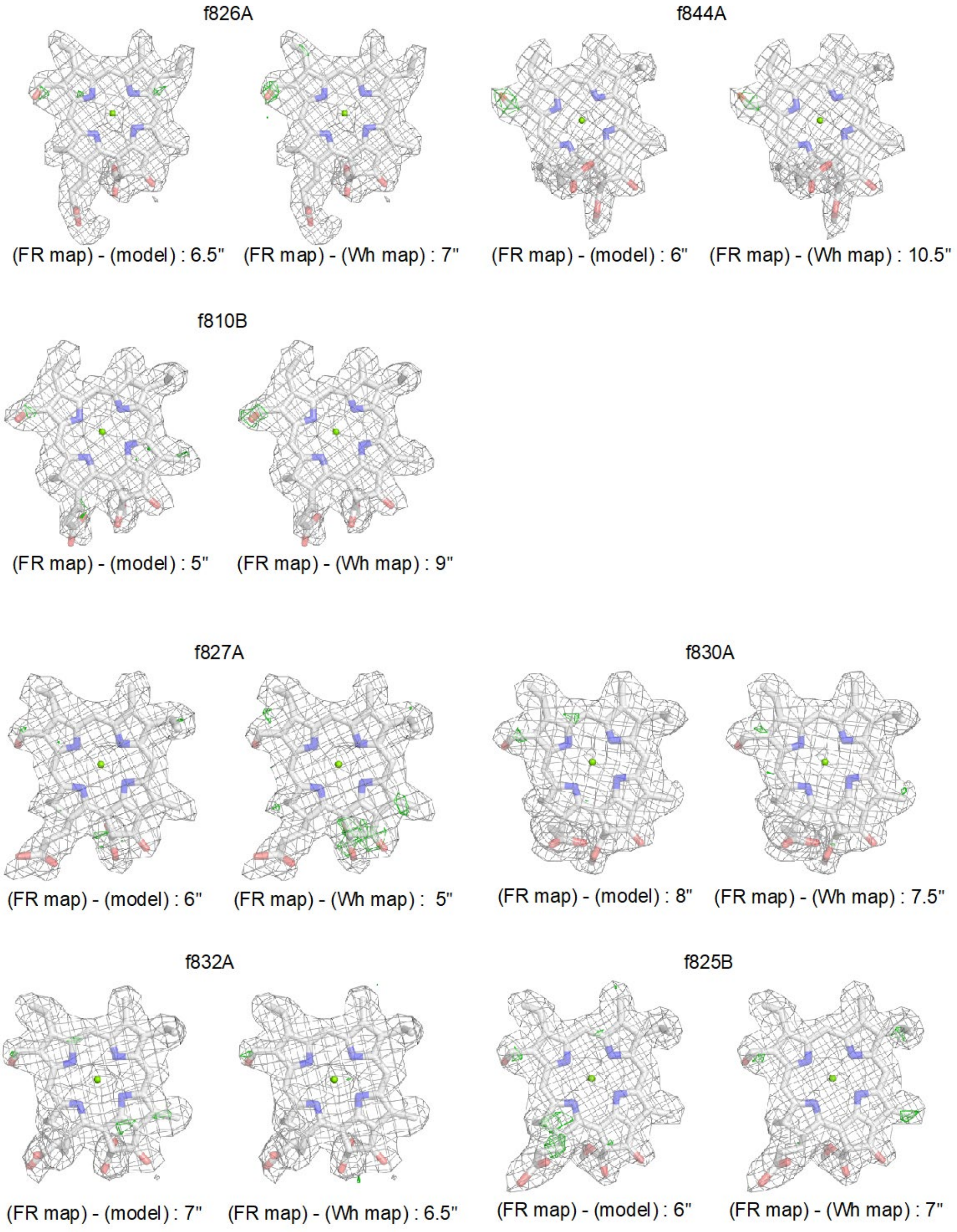

Supplementary Fig. 12. Difference map analysis of Chls *f* in the far-red PSI. The densities for each Chl *f* (f826A, f827A, f830A, f832A, f844A, f810B and f825B) are shown as gray meshes and the corresponding models are shown as gray sticks. Two difference densities (far-red PSI cyro-EM map minus model (left) and far-red PSI cryo-EM map minus white PSI cryo-EM map (right)) for each Chl *f* are shown as green mesh (see Methods).

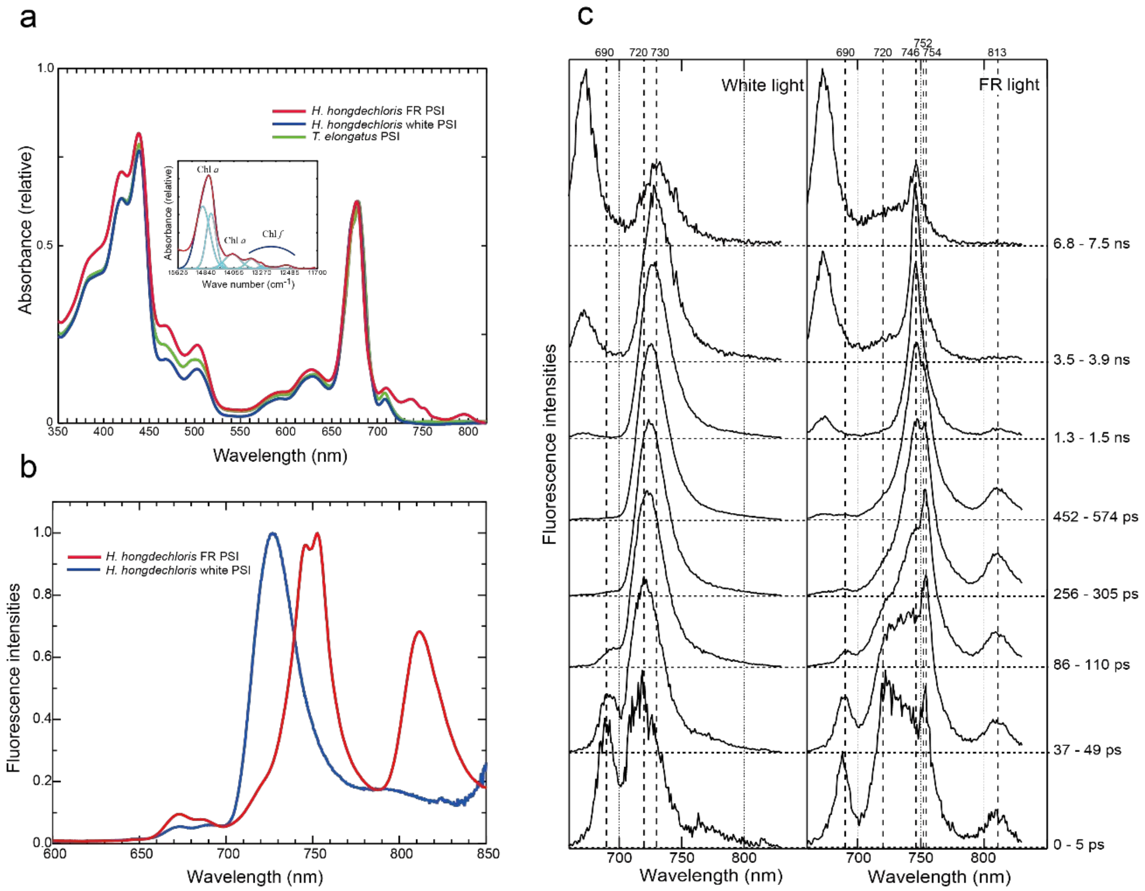

Supplementary Fig. 13. Biochemical and spectroscopic characterizations of the white and far-red PSI. a, Absorption spectra of white PSI, far-red PSI of *H. hongdechloris* and *Thermosynechococcus elongatus* PSI at 80 K. (insert: deconvolution spectrum of the far-red PSI) **b,** Fluorescence spectra of white PSI, far-red PSI of *H. hongdechloris* measured at 77 K. **c,** Time-resolved fluorescence spectra (TRFS) at 77 K. Left side: normalized TRFS,(670–850 nm) of the white PSI. Right side: normalized TRFS (670–850 nm) of the far-red PSI. The individual traces are normalized to their maximum intensities.

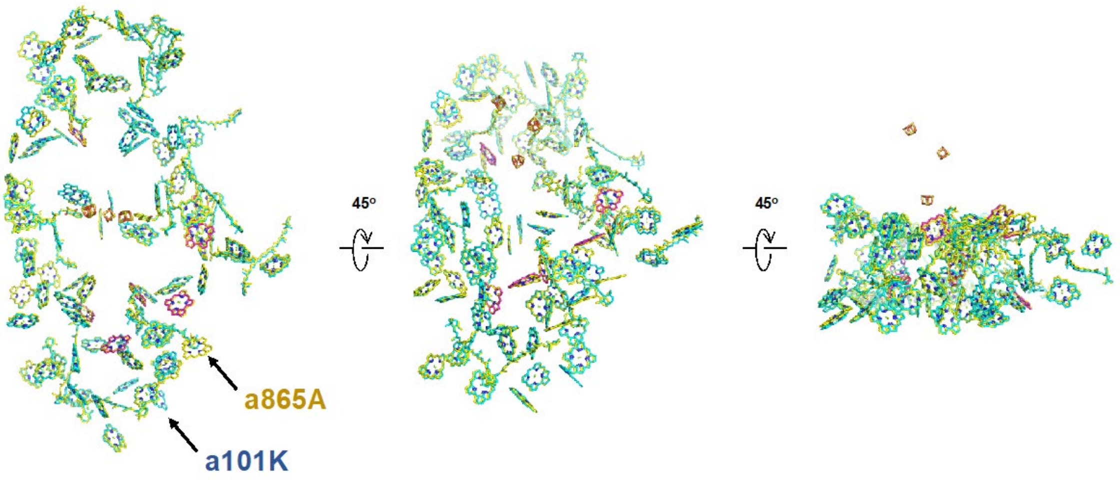

Supplementary Fig. 14. Comparison of pigment orientation between the white PSI monomer and far-red PSI monomer. Pigments of the white PSI monomer (yellow) and far-red PSI monomer (cyan) are superposed with each other. Chls *f* in the far-red PSI are shown as magenta stick.

Supplementary Table 1. Statistics of data collection, processing and refinement.

Complex	White PSI	Far-red PSI
PDB ID	6KMW	6KMX
EMDB ID	EMD-0726	EMD-0727
Data collection and processing		
Microscope	FEI Titan Krios G2	
Detector	Falcon 3EC camera in linear mode	
Nominal magnification	75,000	
Voltage (kV)	300	
Nominal defocus range (μm)	-1.25 to -3.00	
Calibrated pixel size (Å)	0.870	
Total electron dose ($e^-/Å^2$)	47	
Exposure time (s)	2.5	
Number of frames per image	33	
Number of micrographs	4,754	5,712
Initial particle images (no.)	1,224,569	396,129
Final particle images (no.)	546,366	311,993
Map resolution (Å)	2.35	2.41
Applied b-factor ($Å^2$)	-90.3	-90.9
Applied symmetry	C3	C3
Refinement		
Initial Model used (PDB code)	1JB0	1JB0
Model resolution (Å)	2.35	2.41
FSC threshold	0.143	0.143
No. of atoms		
Protein	44,961	46,575
Ligand	18,495	17,901
Water	627	636
B factors ($Å^2$)		
Protein	18.1	24.4
Ligand	23.2	27.8
Water	14.9	17.4
R.m.s deviations		
Bond lengths (Å)	0.008	0.007
Bond angles (°)	1.60	1.57
Validation		
MolProbity score	2.1	1.76
Clashscore	6.35	5.83
Poor rotamers (%)	3.7	2.1
EMRinger score	4.5	4.8
Ramachandran plot		
Favored (%)	95.62	96.82
Allowd (%)	4.27	3.13
Disallowed (%)	0.11	0.05

Supplementary Table 2. Subunit identification by mass spectrometry and N-terminal sequencing.

The band numbers correspond to those labeled in Supplemengary Fig. 1d

Bands*	Mascot Score	Locus tag	Number of matched peptides	N-terminal sequences	Assigned subunits**
1	1509	XM38_051020	70		PsaA3
	748	XM38_051010	32	ATKFPKFSQDL	PsaB3
2	6155	XM38_006600	241	AETLTGKTPVFGGST	PsaD
3	3361	XM38_040180	158	TNQVVKPYLDEP	PsaL3
4	1681	XM38_047750	61	DXAGLTXCS	PsaF2+
5	656	XM38_008840	24	VQRGSKVRILRPESY	PsaE
	44	XM38_031380	2	SHSVKIYDTCIGCTQ	PsaC
6	not detected			AVPPTLEWSPKVAVV	PsaK
7	3067	XM38_010910	121	XXDJHADAH	PsaA1
	2792	XM38_010920	102	ATKFPKFSQDL	PsaB1
8	3873	XM38_010930	114	XGDTHLVP	PsaL1
9	6762	XM38_006600	263	AETLTGKTPVFGGST	PsaD
10	403	XM38_010950	11	XGDTHLVP	PsaF1
11	2256	XM38_008840	89	VQRGSKVRILRPESY	PsaE
	66	XM38_031380	3	SHSVKIYDTCIGCTQ	PsaC
12	not detected			AVPPTLEXSPXVAVV	PsaK

**PsaJ and PsaI were not detected by mass spectrometry and assigned based on their molecular masses and electrophoretic mobilities.

Supplementary Table 3. Sequence identities among four PSI subunits that are expressed from different genes between white and far-red light conditions from three species of cyanobacteria. The species of cyanobacteria listed in the Table are the same as those in Supplementary Figs. 8-11. Average identities are highlighted in grey and yellow.

PsaA	1	2	3	Average	4	5	6	Average
1		86.2	81.8	84.0	74.6	75.4	73.5	74.5
2	86.2		85.3	85.8	74.0	79.3	76.6	76.6
3	81.8	85.3		83.6	75.8	78.3	78.7	77.6
				84.4				76.2
4						83.6	80.1	81.9
5					83.6		85.9	84.8
6					80.1	85.9		83.0
								83.2
PsaB	1	2	3		4	5	6	
1		83.5	80.7	82.1	80.9	81.1	78.9	80.3
2	83.5		87.6	85.6	80.9	85.2	79.9	82.0
3	80.7	87.6		84.2	80.4	82.4	82.6	81.8
				83.9				81.4
4						88.4	84.3	86.4
5					88.4		88.1	88.3
6					84.3	88.1		86.2
								86.9
PsaI	1	2	3		4	5	6	
1		61.8	70.6	66.2	36.8	36.8	35.9	36.5
2	61.8		62.7	62.3	39.5	36.8	35.9	37.4
3	70.6	62.7		66.7	47.4	42.1	35.9	41.8
				65.0				38.6
4						44.7	39.5	42.1
5					44.7		71.1	57.9
6					39.5	71.1		55.3
								51.8
PsaL	1	2	3		4	5	6	
1		72.5	67.8	70.2	43.4	36.3	32.8	37.5
2	72.5		69.9	71.2	41.5	34.6	31.6	35.9
3	67.8	69.9		68.9	43.4	36.3	32.2	37.3
				70.1				36.9
4						36.5	33.3	34.9
5					36.5		50.8	43.7
6					33.3	50.8		42.1
								40.2

Supplementary Table 4. Correspondence of numbering of pigments described in the

text with those in the PDB file for the white and far-red PSI.

	PSI	White	Far-red
**Subunits**	**Chls in the text**	**PDB No. (Chain ID)**	**PDB No. (Chain ID)**
**PsaA**	826	824 (aA, bA, cA)	826 (aA, bA, cA)
	827	825 (aA, bA, cA)	827 (aA, bA, cA)
	829	827 (aA, bA, cA)	829 (aA, bA, cA)
	830	828 (aA, bA, cA)	830 (aA, bA, cA)
	832	830 (aA, bA, cA)	832 (aA, bA, cA)
	835	833 (aA, bA, cA)	835 (aA, bA, cA)
	839	837 (aA, bA, cA)	839 (aA, bA, cA)
	840	838 (aA, bA, cA)	840 (aA, bA, cA)
	841	839 (aA, bA, cA)	841 (aA, bA, cA)
	842	840 (aA, bA, cA)	842 (aA, bA, cA)
	844	840 (aA, bA, cA)	844 (aA, bA, cA)
	865	854 (aA, bA, cA)	-
**PsaB**	810	202 (aL, bL, cL)	202 (aL, bL, cL)
	825	826 (aB, bB, cB)	824 (aB, bB, cB)
	834	834 (aB, bB, cB)	833 (aB, bB, cB)
	835	835 (aB, bB, cB)	834 (aB, bB, cB)
	836	836 (aB, bB, cB)	835 (aB, bB, cB)
	840	840 (aB, bB, cB)	839 (aB, bB, cB)
**PsaK**	101	-	103 (aK, bK, cK)
	**Carotenoids in the text**		
**PsaB**	847	846 (aB, bB, cB)	845 (aB, bB, cB)
**PsaI**	101	101 (aI, bI), 102 (cI)	101 (aI, bI), 103 (cI)
	102	102 (aI, bI), 103 (cI)	203 (aL, bL, cL)

Supplementary Table 5. Cofactors in each monomer of the white and far-red PSI trimers identified in the present study.

Protein		Chlorophyll	Carotenoid	Lipid	Others
PsaA	White PSI	46 Chl *a*	6 BCR	2 LHG	1 [4Fe-4S] cluster, 1 phylloquinone
	Far-red PSI	40 Chl *a*<br>5 Chl *f*	6 BCR	1 LHG	1 [4Fe-4S] cluster, 1 phylloquinone
	TE PSI	46 Chl *a*	6 BCR	2 LHG	1 [4Fe-4S] cluster, 1 phylloquinone
PsaB	White PSI	41 Chl *a*	6 BCR	1 LMG	1 phylloquinone
	Far-red PSI	39 Chl *a*<br>2 Chl *f*	6 BCR	1 LMG	1 phylloquinone
	TE PSI	41 Chl *a*	7 BCR	1 LMG<br>1 LHG	1 phylloquinone
PsaC	White PSI				2 [4Fe-4S] cluster
	Far-red PSI				2 [4Fe-4S] cluster
	TE PSI				2 [4Fe-4S] cluster
PsaD	White PSI				
	Far-red PSI				
	TE PSI				
PsaE	White PSI				
	Far-red PSI				
	TE PSI				
PsaF*	White PSI	-	-	-	-
	Far-red PSI	-	-	-	-
	TE PSI	1 Chl *a*	1 BCR		
PsaI	White PSI		2 BCR		
	Far-red PSI		2 BCR		
	TE PSI		2 BCR		
PsaJ*	White PSI	-	-	-	-
	Far-red PSI	-	-	-	-
	TE PSI	2 Chl *a*	3 BCR		

PsaK*	White PSI	-	-	-	-
	Far-red PSI	1 Chl *a*			
	TE PSI	1 Chl *a*			
PsaL	White PSI	3 Chl *a*	2 BCR		
	Far-red PSI	3 Chl *a*	2 BCR		
	TE PSI	3 Chl *a*	2 BCR		
PsaM	White PSI		1 BCR		
	Far-red PSI		1 BCR		
	TE PSI	1 Chl *a*	1 BCR		
PsbX*	White PSI	-	-	-	-
	Far-red PSI	-	-	-	-
	TE PSI	1 Chl *a*			
Total	White PSI	90	16	3	5
	Far-red PSI	90	16	2	5
	TE PSI	96	22	5	5

BCR, β-carotene; LMG, distearoylmonogalactosyl diglyceride; LHG, dipalmitoylphosphatidyl glycerol; DGD, digalactosyldiacyl glycerol.
*PsaF, PsaJ, PsaK and PsaX in the white PSI and PsaF, PsaJ and PsaX in the far-red PSI are not found in the density.
